# Efficacy and safety of Salvia miltiorrhiza and ligustrazine injection for heart failure: a systematic review and meta-analysis

**DOI:** 10.3389/fphar.2026.1692629

**Published:** 2026-02-09

**Authors:** Pingping Huang, Gaocan Ren, Yifei Wang, Yicheng Liu, Hongwei Zhang, Jiacong Wang, Jinhui Zhang, Yuan Zheng, Lijun Guo, Xiaochang Ma

**Affiliations:** 1 Xiyuan Hospital, China Academy of Chinese Medical Sciences, Beijing, China; 2 Graduate School, China Academy of Chinese Medical Sciences, Beijing, China; 3 Xiyuan Hospital, Beijing University of Chinese Medicine, Beijing, China; 4 National Clinical Research Center for Chinese Medicine Cardiology, Beijing, China

**Keywords:** salvia miltiorrhiza and ligustrazine injection, heart failure, systematic review, meta-analysis, RCTs

## Abstract

**Objective:**

To systematically evaluate the efficacy and safety of SMLI in patients with HF.

**Materials and methods:**

Eight electronic databases were systematically searched from inception up to July 2025 to identify randomized controlled trials (RCTs) comparing SMLI combined with conventional treatment versus conventional treatment alone in patients with HF. Two independent reviewers conducted study selection, data extraction, and risk of bias assessment. Meta-analysis was performed to synthesize efficacy and safety outcomes. Sensitivity analysis was conducted to verify the robustness of the findings, and publication bias was evaluated using funnel plots and Egger’s test.

**Results:**

A total of 32 RCTs involving 3,077 patients were ultimately included. SMLI significantly improved LVEF (MD = 5.62, 95% CI: 4.42–6.81, *P* < 0.01), reduced LVEDD (MD = -5.69, 95% CI:-7.46 to-3.92, *P* < 0.01), and decreased BNP and NT-proBNP levels. It also ameliorated inflammatory markers (CRP, IL-6, TNF-α), endothelial function (ET-1, NO), and increased 6-min walk distance (MD = 60.7, *P* < 0.01). SMLI reduced cardiovascular adverse events (RR = 0.55, *P* < 0.01) without increasing overall adverse reactions.

**Conclusion:**

Combining SMLI with conventional therapy enhances cardiac function and improves clinical outcomes in patients with HF. However, additional high-quality, large-scale RCTs with long-term follow-up are required to validate the long-term efficacy and safety of SMLI, given the potential limitations of the included studies.

**Systematic Review Registration:**

Identifier CRD420251114385.

## 1 Introduction

Heart failure (HF) is a complex, progressive clinical syndrome that constitutes a substantial global public health burden (Triposkiadis et al., 2022). It is characterized by the heart’s impaired ability to pump blood adequately, leading to diverse symptoms, high hospitalization rates, and poor long-term prognosis (including mortality; Guo et al., 2025). Notably, HF is not a single disease but the terminal manifestation of multiple cardiac and non-cardiac conditions, rendering it a multifaceted and clinically challenging disorder to manage (Caturano et al., 2022). Epidemiologically, HF is a highly prevalent condition worldwide. According to the latest data from the Global Burden of Disease Study (GBD), from 1990 to 2021, the global burden of HF—measured by prevalence and years lived with disability (YLD)—increased substantially. The age-standardized prevalence rate rose from 641.14 to 676.68 per 100,000 population, while global HF prevalence more than doubled, increasing from 25.43 million (95% uncertainty interval [UI]: 22.32–29.21 million) to 55.50 million cases (95% UI: 49.00–63.84 million; Ran et al., 2025). In the United States, a recent study estimated that approximately 6.7 million older adults (≥65 years) were diagnosed with HF over the past two decades. Projections suggest this number will rise to 8.7 million by 2030, 10.3 million by 2040, and 11.4 million by 2050 (Bozkurt et al., 2025). Additionally, the rising incidence of comorbidities—including hypertension, diabetes mellitus, and coronary artery disease—further amplifies the global HF burden (Emmons-Bell et al., 2022; Savarese et al., 2023). The core pathophysiological mechanisms of HF include neuroendocrine overactivation (Manolis et al., 2023), chronic inflammation (Wang Y. et al., 2023), vascular endothelial dysfunction (Katz et al., 2005), and left ventricular remodeling (Stencel et al., 2023). These processes perpetuate a vicious cycle that accelerates the decline in cardiac function.

Traditional Chinese Medicine (TCM) has been utilized in HF management for centuries, guided by the theoretical principles of “activating blood circulation to resolve blood stasis” and“nourishing qi to reinforce cardiac function”. Salvia miltiorrhiza (Danshen) and ligustrazine (Chuanxiongqin)—two classic TCM agents for HF—exert multiple cardioprotective effects (Lian et al., 2023; Wang et al., 2020). Salvia miltiorrhiza contains salvianolic acids (e.g., salvianolic acid A, SAA) and tanshinones, which possess anti-inflammatory, antioxidant (Zhang Z. et al., 2023), and anti-fibrotic properties (Lei et al., 2023). Ligustrazine (tetramethylpyrazine, TMP) can reduce ventricular dysfunction and improve cardiac contractility (Lian et al., 2023). Furthermore, a recent preclinical study demonstrated that a Danshensu/tetramethylpyrazine derivative ameliorates cardiac dysfunction and myocardial injury in a rat model of post-myocardial infarction HF by inhibiting matrix metalloproteinase-3 (MMP-3) or β-catenin (Huang et al., 2025). Salvia miltiorrhiza and Ligustrazine Injection (SMLI)—a fixed combination of these two components—has been clinically applied for treating cardiovascular conditions, including coronary heart disease and HF. Emerging small-scale randomized controlled trials (RCTs) have reported that adjuvant SMLI (added to standard Western therapy) may improve left ventricular ejection fraction (LVEF) and reduce B-type natriuretic peptide (BNP) levels in patients with HF (Li et al., 2024; Lin, 2024). However, these studies have notable limitations: small sample sizes, variable dosage regimens, and the absence of assessments for key long-term prognostic indicators (e.g., all-cause mortality, HF readmission rates).

To address these evidence gaps, we performed a comprehensive systematic review and meta-analysis of RCTs to evaluate the efficacy and safety of SMLI for HF treatment. The primary objectives were to: (1) quantify SMLI’s effects on cardiac function (assessed via echocardiographic parameters and BNP/NT-proBNP levels); (2) explore its regulatory effects on key pathophysiological pathways (inflammation, neuroendocrine activation, and vascular endothelial function); (3) evaluate its impact on clinical outcomes (6-min walk test distance [6MWTD]); and (4) characterize its safety profile. This study was designed to provide robust evidence to guide the optimal clinical use of SMLI in HF management.

## 2 Materials and methods

### 2.1 Search strategy

Two authors independently conducted a comprehensive search for RCTs on the use of SMLI in the context of heart failure. The search covered the period from the inception of each respective database up to July 2025. Eight databases were included: PubMed, Embase, Web of Science, China National Knowledge Infrastructure (CNKI), VIP, Wanfang, Cochrane Library, and Sinomed. There were no language restrictions. Any discrepancies or conflicts that arose during the search process were resolved by a third researcher. The search strategy incorporated both MeSH terms and free-text keywords, including but not limited to: heart failure, CHF, acute heart failure, chronic heart failure, Salvia Ligustrazine, Danshen Chuanxiongqin, Radix Salivae Miltiorrhizae ligustrazine, and danshenchuanxiong. Supplementary Table S1 outlines the comprehensive search methodology. This review was conducted according to a protocol registered on PROSPERO (CRD420251114385).

### 2.2 Study design

This systematic review was restricted to RCTs that met the predefined eligibility criteria.

#### 2.2.1 Patients

Individuals with a confirmed diagnosis of HF.

#### 2.2.2 Interventions

The use of SMLI preparations alone or in combination with conventional therapy in eligible HF patients.

#### 2.2.3 Control group interventions and outcome

Patients in the control group were administered either a placebo or standard conventional drugs, including diuretics, Renin-Angiotensin System Inhibitor (RASI), Mineralocorticoid Receptor Antagonist (MRA), β-blockers, cardiotonic, and vasodilators. Outcome measures were established as follows: Primary outcomes include echocardiographic parameters [left ventricular ejection fraction (LVEF), left ventricular end-diastolic diameter (LVEDD), left ventricular end systolic diameter (LVESD), left ventricular end-systolic volume (LVESV),left ventricular end-diastolic volume (LVEDV), Cardiac Index (CI), stroke volume (SV),fractional shortening (FS) and early-to-late diastolic mitral inflow velocity ratio (E/A)], B-type natriuretic peptide (BNP) or N-terminal pro-BNP (NT-proBNP) levels, 6-min walk test distance (6MWTD), neuroendocrine system indicators [Aldosterone (ALD),Plasma Renin Activity (PRA), Angiotensin II(Ang II)],vascular endothelial function markers [endothelin-1 (ET-1) and nitric oxide (NO)], myocardial injury marker troponin I (TnI), inflammatory markers [interleukin-6 (IL-6), C-reactive protein (CRP),high-sensitivity C-reactive protein (hs-CRP), tumor necrosis factor-alpha (TNF-α)], adverse reaction and cardiovascular adverse events.

#### 2.2.4 Criteria for exclusion

Exclusion of studies was based on the following conditions: (1) studies involving non-human subjects; (2) literature accessible only in abstract form without full-text availability; (3) absence of relevant outcome indicators; (4) duplicate publication of identical clinical data across multiple journals; (5) interventions incorporating other traditional Chinese medicine therapies; (6) non-randomized controlled trials (non-RCTs).

### 2.3 Qualification assessment and data extraction

The data extraction process was carried out independently by two reviewers. To streamline the process, key variables—including first author, publication year, country, study design, sample size, mean age, sex, intervention details, and follow-up duration—were systematically cataloged using a standardized Excel spreadsheet tailored for this review. The risk of bias for each included study was assessed following the Cochrane Handbook for Systematic Reviews, using the recommended risk of bias tool. Conflicting assessments were adjudicated by a senior researcher to obtain a unanimous agreement.

### 2.4 Data statistics and analysis

All analyses were performed using Review Manager (version 5.4) and Stata (version 16). Dichotomous outcomes were expressed as odds ratios (OR), while continuous outcomes were presented as mean difference (MD) or standardized mean difference (SMD), depending on the units of measurement, along with their 95% confidence intervals (CI). Heterogeneity was assessed using chi-square statistics; a random-effects model was applied if I^2^statistic exceeding 50%. Otherwise, a fixed-effect model was used when I^2^was≤50% (Higgins and Thompson, 2002). Sensitivity analysis was performed by sequentially excluding each individual study and recalculating the pooled effect size to assess the stability of the meta-analysis results. A stable result was defined as no significant change in the pooled effect size or 95% CI after excluding any single study. Publication bias was assessed using two complementary methods: funnel plots were visually inspected for asymmetry, which may indicate potential publication bias, and Egger’s test was applied for quantitative evaluation, with a significance level of *P* < 0.05 suggesting the presence of significant publication bias. It should be noted that Egger’s test was only performed for outcomes that included three or more studies.

## 3 Results

### 3.1 Literature search and study selection outcomes

**FIGURE 1:**
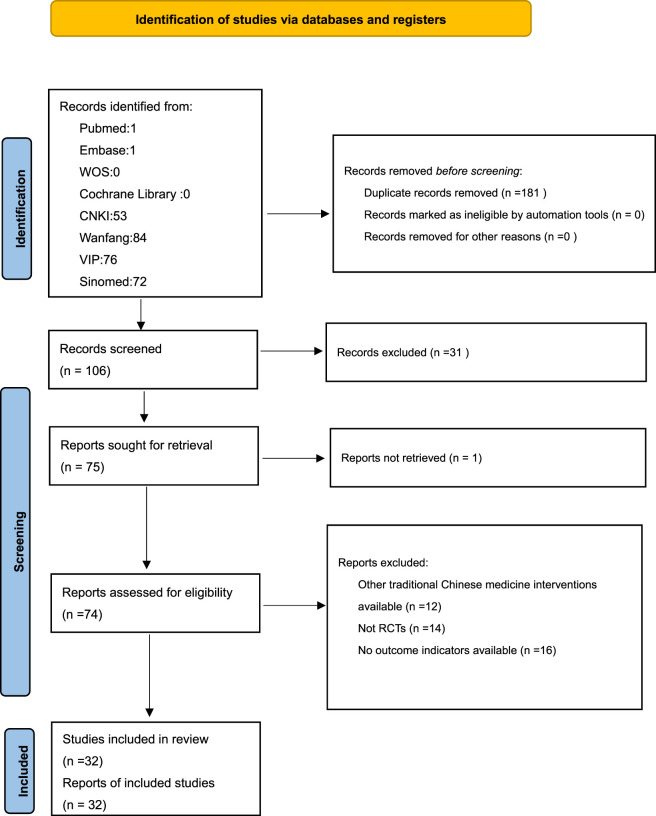
Flowchart for literature search and screening process.

A total of 287 records were identified from the eight literature databases. Following the removal of 181 duplicates, 106 articles were retained for preliminary screening via title and abstract review. From this subset, 31 irrelevant records were excluded, yielding 75 articles for full-text evaluation. One article was excluded due to failure to access the full text or raw data, leaving 74 articles for detailed assessment. Among these 74 articles, 12 were excluded for employing other traditional Chinese medicine interventions, 14 lacked randomization and 16 lacked relevant outcome indicators. Ultimately, 32 articles were included in the analysis. The study selection process is depicted in Figure 1.

### 3.2 Descriptives of analyzed studies

The 32 included studies were exclusively published in Chinese journals between 2010 and 2024. Sample sizes of the randomized controlled trials (RCTs) ranged from 30 to 180 patients with HF, totaling 3,077 participants—comprising 1,535 in the SMLI group and 1,542 in the control group. Treatment duration ranged from 10 days to 6 months. In the SMLI treatment groups, the daily dosage of SMLI varied from 5 mL to 20 mL. Further details are presented in Table 1.

**TABLE 1:** Summary of studies incorporated in the analysis.

Study	Sample size		Male/Female		Age		Classification of NYHA heart function (II/III/IV)		Course of disease (years)		Intervention(s)		Treatment duration	Outcomes
	T	C	T	C	T	C	T	C	T	C	T	C		
Lin (2024)	34	34	19/15	18/16	60.19 ± 9.38	60.23 ± 9.41	11/13/10	10/12/12	3.26 ± 1.01	3.21 ± 1.05	CGT + SMLI (5 mL iv drip qd)	CTs	4W	①②
Li et al. (2024)	55	55	29/26	30/25	62.23 ± 0.66	61.21 ± 0.62	—	—	3.06 ± 0.34	3.01 ± 0.30	CGT + SMLI (5 mL iv drip qd)	CTs	8w	①②⑥⑦⑧
Li (2024)	43	43	22/21	23/20	67.80 ± 5.02	67.85 ± 4.85	20/15/8	20/14/9	4.10 ± 1.73	4.15 ± 1.69	CGT + SMLI (10 mL iv drip qd)	CTs	2W	①②⑤⑧
Zhang J.Y. et al. (2023)	36	35	27/9	24/11	51.50 ± 3.83	50.00 ± 4.25	25/11/NA	22/13/NA	3.25 ± 0.60	3.50 ± 0.43	CGT + SMLI (10 mL iv drip qd)	CTs	2m	①⑥⑦
Sun and Wang (2023)	80	80	45/35	39/41	64.07 ± 6.91	63.89 ± 7.01	NA/45/35	NA/39/41	3.29 ± 0.88	3.34 ± 0.91	CGT + SMLI (10 mL iv drip qd)	CTs	2W	①②⑦⑧
Chen (2023)	45	45	27/18	25/20	65.05 ± 2.78	65.12 ± 2.65	33/12/NA	31/14/NA	1.95 ± 0.44	1.98 ± 0.41	CGT + SMLI (20 mL iv drip qd)	CTs	2W	①
Zhong (2021)	39	39	21/18	20/19	58.72 ± 5.96	58.45 ± 6.27	21/15/3	20/17/2	4.94 ± 1.15	5.05 ± 1.10	CGT + SMLI (10 mL iv drip qd)	CTs	6m	①②③⑥
Huang (2021)	30	30	37/23		52.31 ± 4.4		14/26/30		—	—	CGT + SMLI (10 mL iv drip qd)	CTs	10d	②
(Cui et al., 2021)	75	75	39/36	40/35	60.3 ± 2.6	59.3 ± 2.5	—	—	2.31 ± 1.31	2.51 ± 0.93	CGT + SMLI (20 mL iv drip qd)	CTs	2W	①②⑤⑥⑦
Sun et al. (2020)	39	39	21/18	20/19	58.53 ± 4.95	58.43 ± 4.85	—	—	4.91 ± 2.16	5.04 ± 2.20	CGT + SMLI (10 mL iv drip qd)	CTs	2W	①
Sui et al. (2020)	35	35	32/38		—	—	—	—	—	—	CGT + SMLI (10 mL iv drip qd)	CTs	2W	①②
Shi (2020)	20	20	24/32		59.78 ± 5.61		19/17/20		—	—	CGT + SMLI (5–10 mL iv drip qd)	CTs	2W	①
Mai et al. (2020)	75	75	41/34	39/36	52.24 ± 5.12	52.17 ± 5.46	19/19/13	20/21/12	—	—	CGT + SMLI	CTs	2W	①
Gao (2020)	75	75	46/29	42/33	58.0 ± 4.4	58.2 ± 4.2	23/32/20	26/30/19	4.4 ± 2.7	4.7 ± 2.4	CGT + SMLI (10 mL iv drip qd)	CTs	2W	①⑥
Cai et al. (2020)	50	50	28/22	26/24	72.48 ± 5.13	72.45 ± 5.12	16/15/19	15/17/18	10.31 ± 2.18	10.23 ± 2.15	CGT + SMLI (10 mL iv drip qd)	CTs	2W	①②⑥⑧
Zhao et al. (2019)	60	60	39/21	37/23	61.13 ± 4.57	59.73 ± 4.35	18/21/21	21/19/20	—	—	CGT + SMLI (10 mL iv drip qd)	CTs	2W	①②⑤⑥
Yang et al. (2019)	40	40	27/13	29/11	66.8 ± 6.9	67.5 ± 5.4	20/14/6	22/13/5	—	—	CGT + SMLI (10 mL iv drip qd)	CTs	10-14d	①⑥⑧⑨
Zhang et al. (2018)	50	50	26/24	28/22	76.1 ± 5.3	75.2 ± 6.1	—	—	—	—	CGT + SMLI (10 mL iv drip qd)	CTs	2W	①②③
Zhang (2018)	66	66	36/30	34/32	65.1 ± 10.6	64.8 ± 9.5	18/35/13	21/33/12	15.7 ± 4.2	17.9 ± 4.5	CGT + SMLI (40 mg iv drip qd)	CTs	2W	①②⑦
Han et al. (2017)	43	43	25/18	23/20	57.2 ± 8.3	58.3 ± 9.2	16/17/10	18/15/10	11 ± 4	10 ± 5	CGT + SMLI (10 mL iv drip qd)	CTs	2W	①⑧
Bi et al. (2017)	40	40	23/17	21/19	68.5 ± 18.5	69.0 ± 23.2	—	—	—	—	CGT + SMLI (10 mL iv drip qd)	CTs	2W	①②⑧
Shi (2016)	80	100	48/32	60/40	66士3	66 ± 4	49/31/NA	59/41/NA	5.71 ± 1.8	5.9 ± 2.3	CGT + SMLI (10 mL iv drip qd)	CTs	2W	①
Shao (2016)	35	35	42/28		69.7 ± 7.4		13/46/11		12.6 ± 4.2		CGT + SMLI (5–10 mL iv drip qd)	CTs	2W	①④⑨
Qin et al. (2016)	46	46	58/34		69.5 ± 6.6		18/54/20		12.7 ± 3.5		CGT + SMLI (10 mL iv drip qd)	CTs	10d	②
Han et al. (2016)	75	75	50/25	43/32	66 ± 4	68 ± 5	—	—	—	—	CGT + SMLI (10 mL iv drip qd)	CTs	2W	①②
Wang (2015)	54	50	34/20	32/18	41.4 ± 9.2	40.5 ± 9.4	NA/22/32	NA/20/30	—	—	CGT + SMLI (5 mL iv drip qd)	CTs	4W	①
Cui et al. (2015)	15	15	9/6	10/5	—	—	—	—	—	—	CGT + SMLI (10 mL iv drip qd)	CTs	2W	②
Yu and Liu (2014)	34	34	20/14	20/14	59.3 ± 7.4	59.2 ± 7.6	—	—	—	—	CGT + SMLI (10 mL iv drip qd)	CTs	2W	①④⑥
Gu (2013)	52	50	53/49		—	—	18/40/44		—	—	CGT + SMLI (20 mL iv drip qd)	CTs	2W	①②
Zhang (2011)	32	30	18/14	17/13	63.8	62.8	6/12/14	5/12/13	​	​	CGT + SMLI (10 mL iv drip qd)	CTs	2W	①
Lu and Wu (2011)	51	45	54/42		66.5		17/38/41		—	—	CGT + SMLI (10 mL iv drip qd)	CTs	2W	①
Zhuang (2010)	31	33	20/21	17/16	57 ± 11.2	58 ± 10.4	NA/18/15	NA/17/14	13.2 ± 6.1	13.0 ± 5.7	CGT + SMLI (10 mL iv drip bid)	CTs	1W	①

T, intervention group; C, control group; CGT, control group treatment; CTs, conventional treatments; ① echocardiography; ② B-type natriuretic peptide/N-terminal pro-BNP, levels; ③Neuroendocrine system indicators; ④ 6-min walk test distance; ⑤Myocardial injury indicators ⑥ Inflammatory indicators; ⑦vascular endothelial function markers; ⑧ adverse reaction; ⑨cardiovascular adverse events.

**FIGURE 2:**
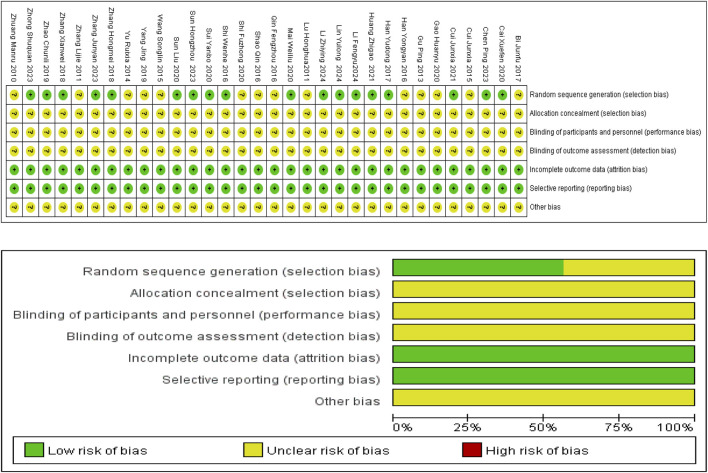
Risk of bias assessment for included studies.

Among the included studies, 18 implemented randomization via either the random number table method or random sampling method, and were assessed as having low bias risk. The remaining 14 only stated that random grouping was used without specifying the exact randomization method, leading to unclear bias risk in this domain. All studies failed to report allocation concealment, resulting in unclear bias risk for this item. Additionally, none of the studies described blinding procedures for researchers, participants, or outcome assessors, resulting in unclear bias risk for blinding. All included RCTs reported complete outcome data, indicating low reporting bias risk. No obvious evidence of other potential biases was identified across the studies. However, the absence of detailed descriptions of relevant information meant the risk of such biases was judged to be unclear. Results of the bias risk assessment are presented in Figure 2, with the assessment process strictly adhering to guidelines outlined in the Cochrane Handbook for Systematic Reviews.

### 3.3 Primary outcomes from meta-analysis

#### 3.3.1 Echocardiographic indicators

##### 3.3.1.1 LVEF

**FIGURE 3:**
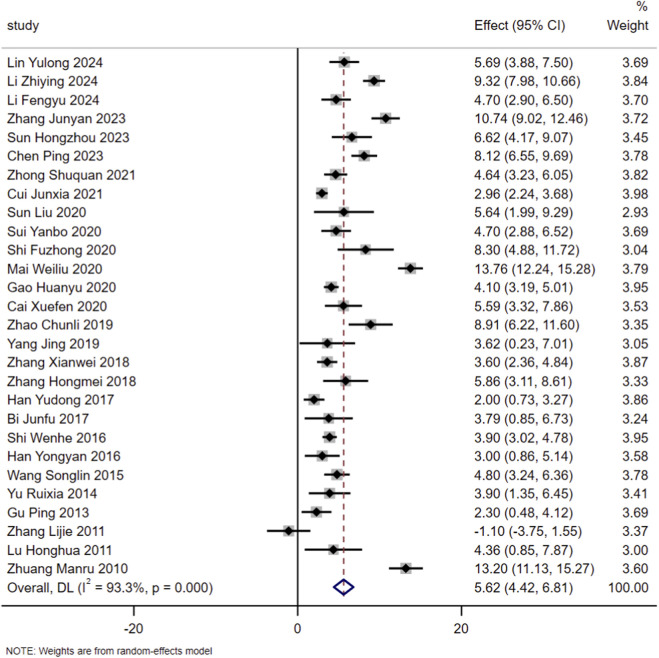
Forest plot for LVEF.

A total of 28 eligible studies were included in the present meta-analysis. Heterogeneity assessment demonstrated substantial heterogeneity across the included studies (I^2^ = 93.3%, *P* < 0.01), prompting the adoption of a random-effects model for pooled analysis. Results indicated that in addition to standardized treatment for patients with HF, the administration of SMLI significantly improved LVEF (MD = 5.62, 95% CI: 4.42–6.81, *P* < 0.01). Detailed information on the above results can be found in Figure 3.

##### 3.3.1.2 LVEDD

**FIGURE 4:**
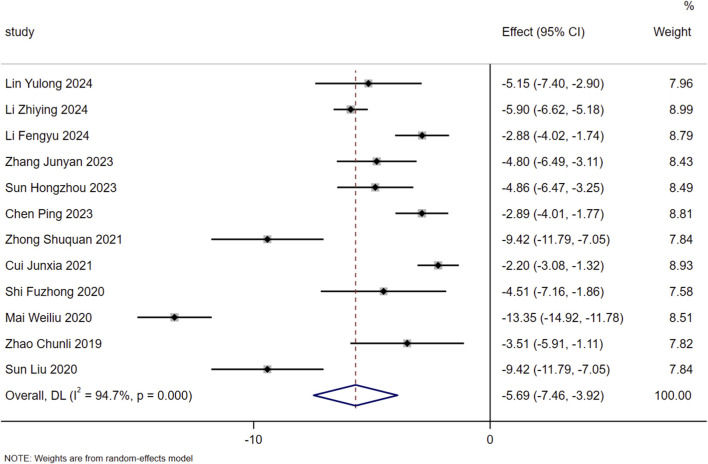
Forest plot for LVEDD.

A total of 12 studies meeting the inclusion criteria were included in this meta-analysis. Heterogeneity assessment demonstrated substantial heterogeneity across the included studies (I^2^ = 94.7%, *P* < 0.01), therefore a random-effects model was employed for data pooling in the present study. The results indicated that on the basis of standardized treatment for patients with HF, the additional administration of SMLI could significantly improve the LVEDD of patients, with a statistically significant difference (MD = −5.69, 95% CI: −7.46 to −3.92, *P* < 0.01). Detailed information on the above results can be found in Figure 4.

**FIGURE 5:**
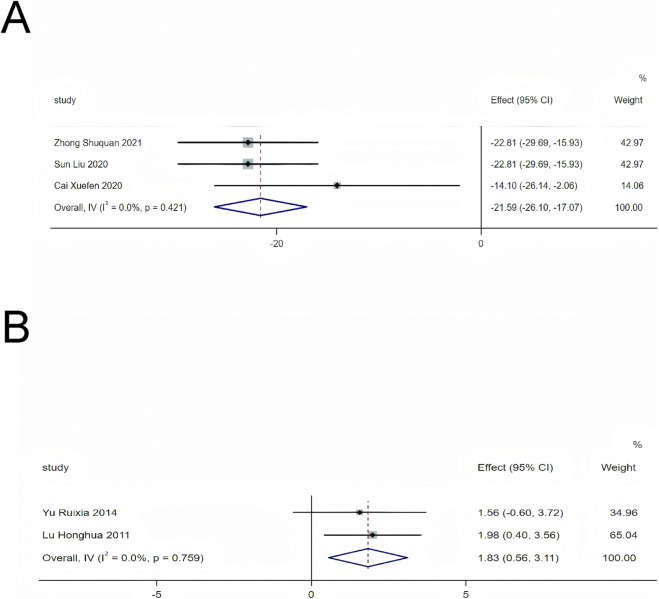
Forest plot for LVESV and FS. **(A)** LVESV; **(B)** FS.

For other echocardiographic indicators, we found that standardized treatment with SMLI combined with HF significantly reduced LVESV levels (MD = −21.59, 95% CI: −26.10 to −17.07, *P* < 0.01) and FS levels (MD = 1.83, 95% CI: 0.56 to 3.11, *P* < 0.01). Please refer to Figure 5 for specific details. In addition, standardized treatment of SMLI combined with HF can significantly reduce LVESD (MD = −4.72, 95% CI: 6.34 to −3.12, *P* < 0.01)、LVESV(MD = −13.25, 95% CI: 23.9 to −2.6, *P* = 0.01), improve CI(MD = 0.45, 95% CI:0.04 to 0.86, *P* = 0.03),SV(MD = 5.80, 95% CI:0.95 to 10.64, *P* = 0.02) and E/A (MD = 0.21, 95% CI: 0.12 to 0.3, *P* < 0.01) levels, specific content can refer to Supplementary Material Supplementary Figure S1.

#### 3.3.2 BNP and NTproBNP

**FIGURE 6:**
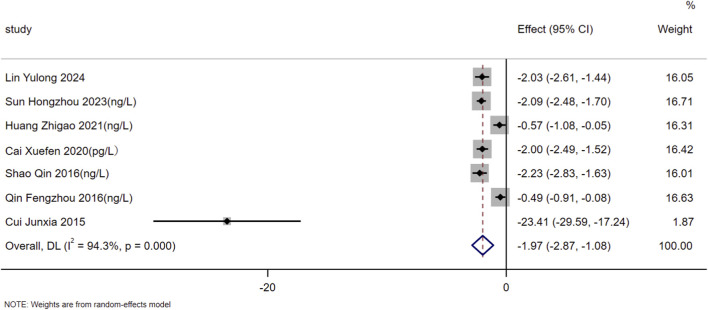
Forest plot for BNP.

**FIGURE 7:**
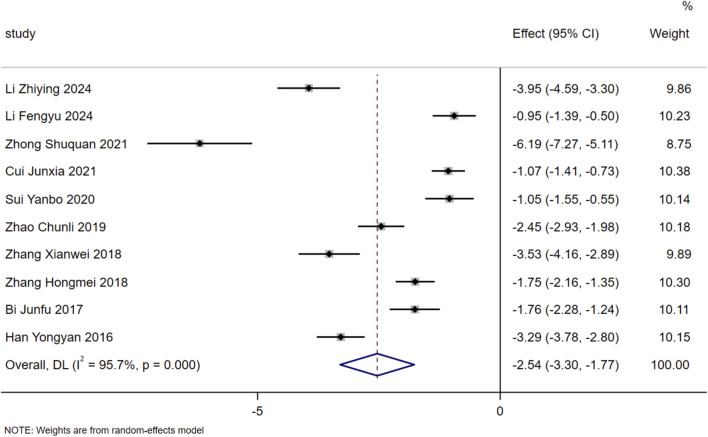
Forest plot for NT-proBNP.

Regarding the biomarkers of BNP and NT-proBNP, relevant detection data were reported in 7 and 10 of the included studies, respectively. Heterogeneity assessment revealed substantial heterogeneity among the included studies for both indicators (BNP: I^2^ = 94.3%, *P* < 0.01; NT-proBNP: I^2^ = 95.7%, *P* < 0.01). Thus, a random-effects model was used for data pooling in this study. The results demonstrated that on the basis of standardized treatment for patients with HF, the addition of SMLI could significantly reduce the levels of BNP (SMD = −1.97, 95% CI: −2.87 to −1.08, *P* < 0.01) and NT-proBNP (SMD = −2.54, 95% CI: −3.30 to −1.77, *P* < 0.01) in patients. Detailed results are presented in Figures 6, 7.

#### 3.3.3 Neuroendocrine system indicators

**FIGURE 8:**
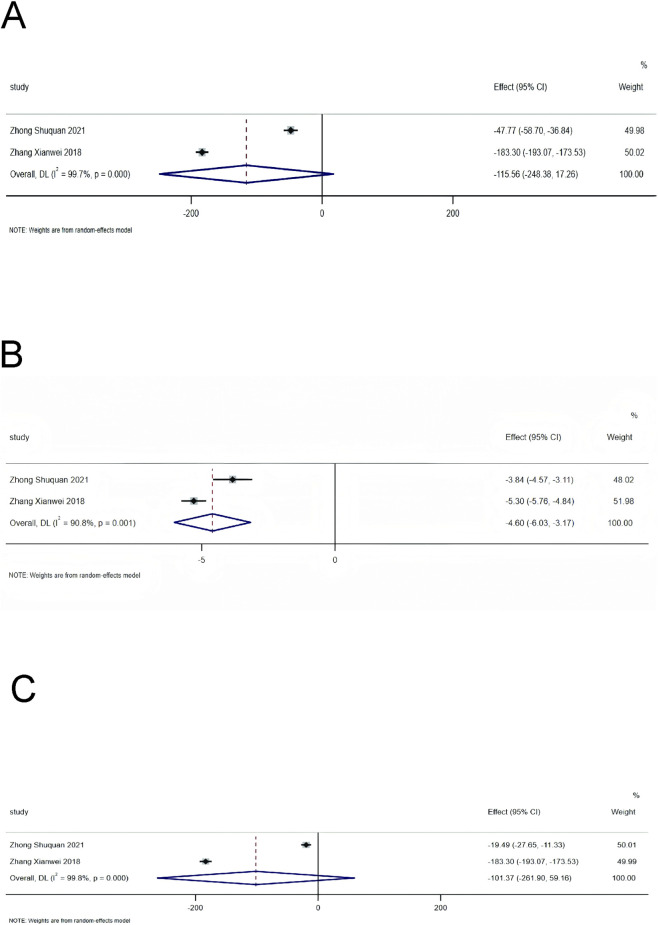
Forest plot for neuroendocrine system indicators. **(A)** ALD; **(B)** PRA; **(C)** Ang-II.

The neuroendocrine-related indicators included in the analysis of this study were ALD, PRA, and Ang-II. Heterogeneity assessment showed substantial heterogeneity among the included studies for each of the aforementioned indicators, so a random-effects model was adopted for data pooling. The results indicated that on the basis of standardized treatment for patients with HF, the addition of SMLI significantly reduced the level of PRA in patients, with a statistically significant difference (MD = −4.60, 95% CI: −6.03 to −3.17, *P* < 0.01). For the two indicators of ALD and Ang-II, the 95% CI corresponding to their effect sizes crossed the null effect line, which suggested that there was no statistically significant difference between the two groups in terms of these two indicators. The specific data were as follows: ALD (MD = −115.56, 95% CI: −248.38 to 17.26, *P* = 0.09) and Ang-II (MD = −101.37, 95% CI: −261.90 to 59.16, *P* = 0.22). Detailed information on the above results can be found in Figure 8.

#### 3.3.4 Six-minute walking distance

**FIGURE 9:**
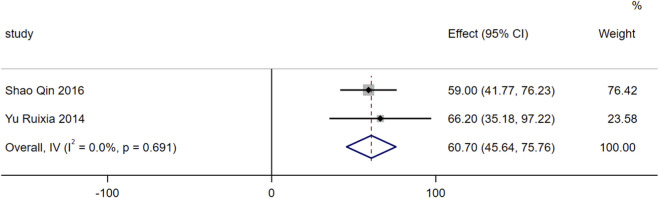
Forest plot for 6MWD.

For the indicator of 6MWD, a total of 2 included studies reported relevant data. Heterogeneity assessment demonstrated low heterogeneity among the included studies for this indicator (I^2^ = 0%, *P* = 0.69). Therefore, a fixed-effects model was used for data pooling. The results showed that on the basis of standardized treatment for patients with HF, the addition of SMLI significantly increased the 6MWD of patients, with a statistically significant difference (MD = 60.7, 95% CI: 45.64 to 75.76, *P* < 0.01). Detailed results are presented in Figure 9.

#### 3.3.5 TnI

**FIGURE 10:**
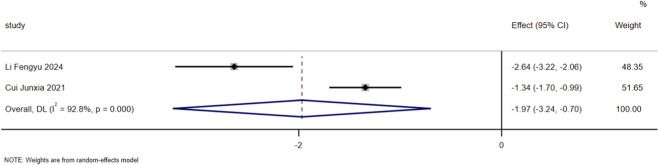
Forest plot for TnI.

For the indicator of TnI, a total of 2 included studies reported relevant detection data. Heterogeneity assessment revealed substantial heterogeneity among the included studies for this indicator (I^2^ = 92.8%, *P* < 0.01). Thus, a random-effects model was employed for data pooling. The results demonstrated that on the basis of standardized treatment for patients with HF, the addition of SMLI significantly reduced the level of TnI in patients, with a statistically significant difference (SMD = −1.97, 95% CI: −3.24 to −0.70, *P* < 0.01). Detailed results are presented in Figure 10.

#### 3.3.6 Inflammatory indicators

**FIGURE 11:**
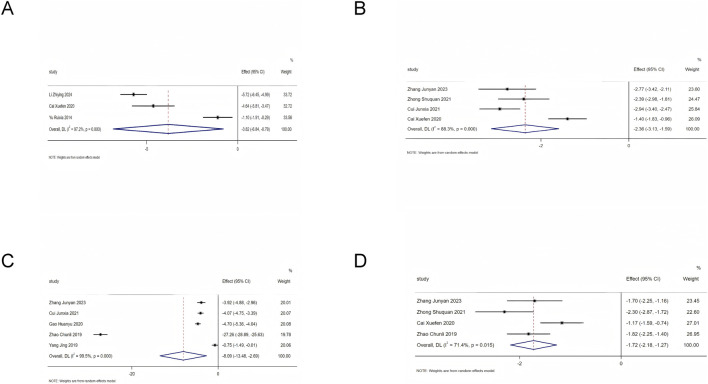
Forest plot for inflammatory indicators. **(A)** CRP; **(B)** IL-6, **(C)** hs-CRP, **(D)** TNF-α.

vThe inflammatory indicators included in the analysis of this study were CRP, Hs-CRP, IL-6, and TNF-α. Heterogeneity assessment showed substantial heterogeneity among the included studies for each indicator, so a random-effects model was adopted for data pooling. The results indicated that on the basis of standardized treatment for patients with HF, the addition of SMLI significantly reduced the levels of CRP (MD = −3.82, 95%CI: −6.84 to −0.79, *P* = 0.01), Hs-CRP (MD = −8.09, 95%CI: −13.48 to −2.69, *P* < 0.01), IL-6 (SMD = −2.36, 95%CI: −3.13 to −1.59, *P* < 0.01), and TNF-α (SMD = −1.72, 95%CI: −2.18 to −1.27, *P* < 0.01) in HF patients. Detailed results are presented in Figure 11.

#### 3.3.7 Vascular endothelial function indicators

**FIGURE 12:**
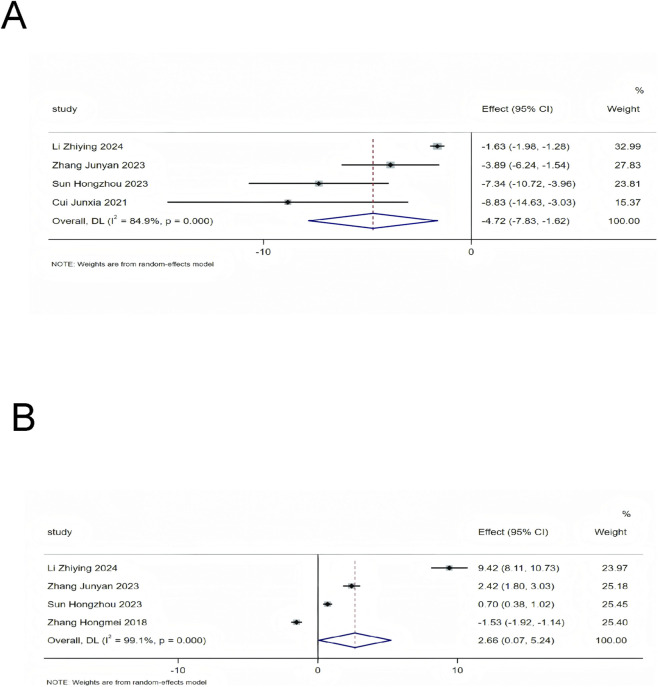
Forest plot for vascular endothelial function indicators. **(A)** ET-1; **(B)** NO.

A total of 4 included studies provided relevant data on ET-1, with the same number of studies reporting data for NO. Heterogeneity assessment revealed substantial heterogeneity among the studies for both indicators (ET-1: I^2^ = 84.9%, *P* < 0.01; NO: I^2^ = 99.1%, *P* < 0.01). Thus, a random-effects model was used for data pooling in this study. The results showed that on the basis of standardized treatment for HF patients, the addition of SMLI significantly reduced ET-1 levels (MD = −4.72, 95%CI: −7.83 to −1.62, *P* < 0.01) and significantly increased NO levels (SMD = 2.66, 95%CI: 0.07 to 5.24, *P* = 0.04) in patients. Detailed results are presented in Figure 12.

#### 3.3.8 Adverse reaction

**FIGURE 13:**
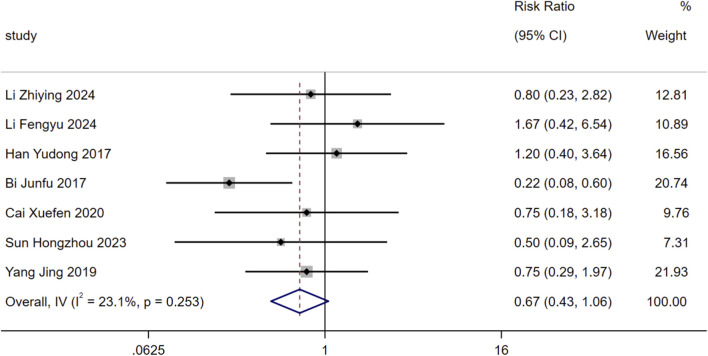
Forest plot for adverse reaction.

Adverse reactions (ARs) reported in the included studies were integrated for analysis, with the main ARs including dizziness and headache, nausea and vomiting, rash, hypotension, fever, insomnia, hyperkalemia, cough, renal injury, angioedema, and fast heart rate. The results of the overall analysis showed that the 95%CI corresponding to the effect size crossed the null effect line of “1” (RR = 0.67, 95%CI: 0.43 to 1.06, *P* = 0.09). This suggested no statistically significant difference in the incidence of ARs between the two groups, indicating that SMLI has a certain degree of safety. Detailed results are presented in Figure 13.

#### 3.3.9 Cardiovascular adverse events

**FIGURE 14:**
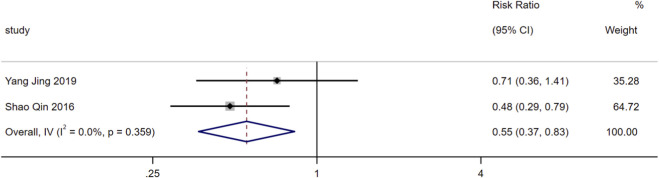
Forest plot for cardiovascular adverse events.

Data on cardiovascular adverse events were reported in 2 included studies. Heterogeneity assessment showed low heterogeneity among the studies for this outcome (I^2^ = 0%, *P* = 0.36). Therefore, a random-effects model was used for analysis in this study. The results demonstrated that on the basis of standardized treatment for HF patients, the addition of SMLI significantly reduced the risk of cardiovascular adverse events (RR = 0.55, 95%CI: 0.37 to 0.83, *P* < 0.01). Detailed results are presented in Figure 14.

### 3.4 Sensitivity analysis and publication bias

**FIGURE 15:**
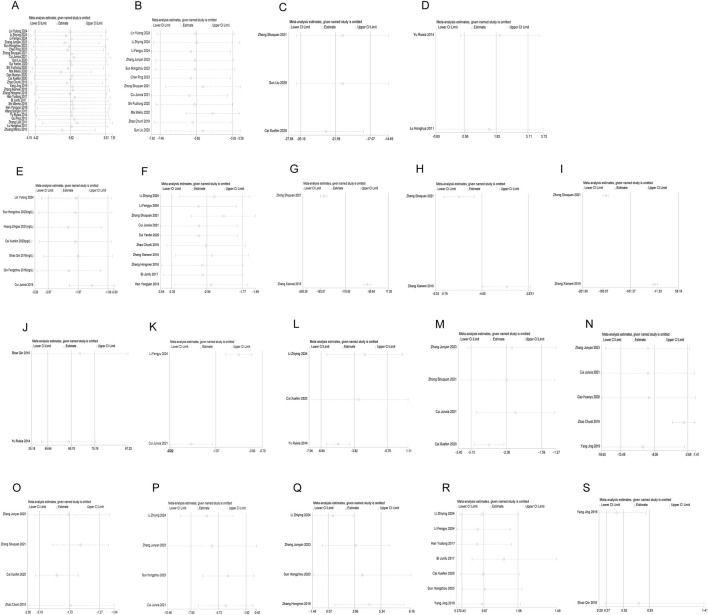
Sensitivity analysis. **(A)** LVEF, **(B)** LVEDD, **(C)** LVESV, **(D)** LVFS, **(E)** BNP, **(F)** NT-proBNP, **(G)** ALD, **(H)** PRA, **(I)** Ang-II, **(J)** 6MWT, **(K)** TnI, **(L)** CRP, **(M)** IL-6, **(N)** hs-CRP, **(O)** TNF-α, **(P)** ET-1, **(Q)** NO, **(R)** Adverse reaction, **(S)** Cardiovascular adverse events.

**FIGURE 16:**
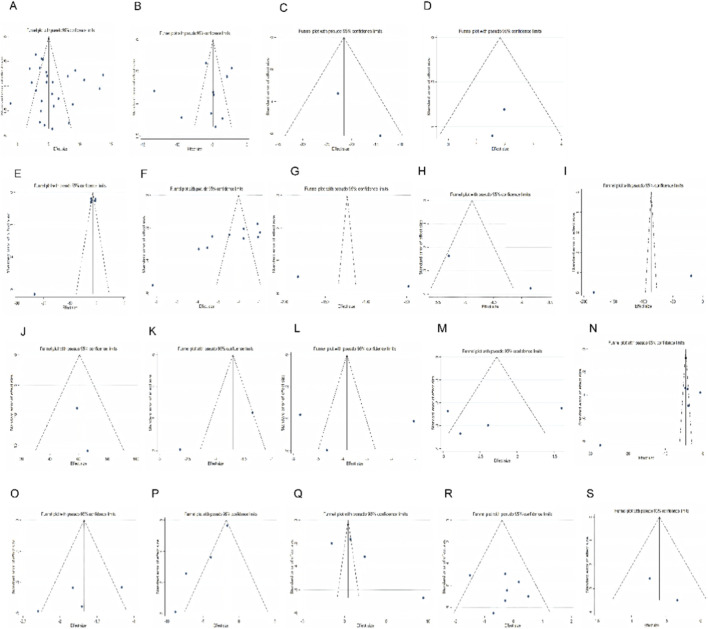
Funnel plot. **(A)** LVEF, **(B)** LVEDD, **(C)** LVESV, **(D)** LVFS, **(E)** BNP, **(F)** NT-proBNP, **(G)** ALD, **(H)** PRA, **(I)** Ang-II, **(J)** 6MWT, **(K)** TnI, **(L)** CRP, **(M)** IL-6, **(N)** hs-CRP, **(O)** TNF-α, **(P)** ET-1, **(Q)** NO, **(R)** Adverse reaction, **(S)** Cardiovascular adverse events.

Sensitivity analysis of the results of all included studies was performed using a one-study-at-a-time exclusion approach. The results showed that excluding any single study did not cause significant changes in the pooled effect size or the stability of conclusions, indicating that the results of this meta-analysis have good reliability (Table 2; Figure 15; Supplementary Figure S2). Meanwhile, funnel plot analysis was conducted for all outcome indicators to assess publication bias. In addition, Egger’s test were performed for outcome indicators with more than 3 included studies to examine publication bias. No publication bias was detected in the analysis of all indicators except NT-proBNP and ET-1. The results of these assessments are detailed in Figure 16; Supplementary Figure S3 and Table 3.

**TABLE 2:** Overview of sensitivity analysis.

Indicators	OR/MD/SMD fluctuations	95%CI fluctuations
LVEF	5.62	(4.42,6.18)
LVEDD	−5.96	(-7.46,-3.92)
LVESV	−21.59	(-26.1,-17.07)
LVFS	1.83	(0.56,3.11)
BNP	−1.97	(-2.87,-1.08)
NT-proBNP	−2.54	(-3.30,-1.77)
ALD	−115.56	(-248.38,17.26)
PRA	−4.6	(-6.03,3.17)
Ang-II	−101.37	(-261.90,59.16)
6MWT	60.7	(45.64,75.76)
TnI	−1.97	(-3.24,-0.7)
CRP	−3.82	(-6.84,-0.79)
IL-6	−2.36	(-3.13,-1.59)
Hs-CRP	−8.09	(-13.48,-2.69)
TNF-α	−1.72	(-2.18,-1.27)
ET-1	−4.72	(-7.83,-1.62)
NO	2.66	(0.07,5.24)
Adverse reaction	0.67	(0.43,1.06)
Cardiovascular adverse events	0.55	(0.37,0.83)

**TABLE 3:** Overview of publication bias.

Indicators	*P* value for Egger’s test
LVEF	0.162
LVDD	0.325
LVESD	0.609
E/A	0.105
SV	0.196
BNP	0.095
NT-proBNP	0.004
CRP	0.966
IL-6	0.545
Hs-CRP	0.079
TNF-α	0.336
ET-1	0.024
NO	0.219
Adverse reaction	0.493

## 4 Discussion

### 4.1 Core findings: summary and clinical interpretation

This study systematically searched 8 databases, ultimately including 32 RCTs involving a total of 3,077 HF patients. For the first time, this study validates the synergistic effect of SMLI combined with conventional treatments in a large sample size. The core results can be interpreted across four dimensions.

#### 4.1.1 Substantial improvement in cardiac function indicators

Echocardiographic results show that SMLI significantly increases LVEF (MD = 5.62, 95% CI: 4.42–6.81, *P* < 0.01) as well as FS (MD = 1.83, 95% CI: 0.56–3.11, *P* < 0.01). Meanwhile, it reduces LVEDD (MD = −5.69, 95% CI: −7.46 to −3.92, P < 0.01) and LVESV (MD = −21.59, 95% CI: −26.10 to −17.07, *P* < 0.01). The method for measuring LVEF was first proposed in the early 1960s by Stuart Bartle, a young cardiovascular researcher at the University of Virginia (Bartle and Sanmarco, 1966). LVEF is the gold standard for assessing systolic function in patients with HF. Currently, LVEF can be used to predict event risk and therapeutic responses in HF (Dimond et al., 2024). An increase of 5.62% in LVEF meets the threshold for meaningful clinical benefit. Furthermore, reductions in LVEDD and LVESV suggest that SMLI can reverse left ventricular remodeling. This finding aligns with a core therapeutic objective in HF management, given that reversing left ventricular remodeling helps delay disease progression.

#### 4.1.2 Regulatory effects on pathophysiological markers

Inhibition of neuroendocrine activation: SMLI significantly reduces PRA (MD = −4.60, 95% CI: −6.03 to −3.17, *P* < 0.01) but exerts no significant effect on ALD or Ang-II (*P* > 0.05). During the pathological progression of HF, reduced cardiac output and insufficient renal perfusion trigger pathological activation of the Renin-Angiotensin-Aldosterone System (RAAS). Increased renin release—characterized by elevated PRA—catalyzes the conversion of angiotensinogen to Ang-I, which is further converted to Ang-II via the action of Angiotensin-Converting Enzyme (ACE). By binding to the AT1 receptor, Ang-II mediates multiple pathophysiological effects that drive HF progression, including systemic vasoconstriction (increasing cardiac afterload), ALD release from the adrenal cortex (promoting sodium and water retention to exacerbate cardiac preload), direct induction of myocardial fibrosis, and excessive sympathetic activation (Koba, 2018). Previous studies confirm that inhibiting RAAS activation significantly reduces mortality risk in patients with HF (Ji et al., 2023). Accordingly, measuring RAAS-related indicators provides a valuable approach to evaluating the therapeutic efficacy of HF treatments. Currently, there is a paucity of literature reporting on RAAS-related indicators, which may explain the lack of statistical significance in ALD and Ang-II outcomes in the present study.

Alleviation of inflammation and myocardial injury: SMLI significantly reduces CRP (MD = −3.82, *P* = 0.01), IL-6 (SMD = −2.36, *P* < 0.01), TNF-α (SMD = −1.72, *P* < 0.01), and TnI (SMD = −1.97, *P* < 0.01). A key pathological basis of HF is a “chronic inflammatory state”: elevated inflammatory factors exacerbate myocardial cell necrosis and fibrosis. The anti-inflammatory effect of SMLI may represent an important intermediate mechanism underlying its improvement of cardiac function. Proinflammatory cytokines—including TNF-α, IL-1, and IL-6—are widely implicated in HF pathogenesis (Hanna and Frangogiannis, 2020). These cytokines regulate the phenotype and function of all cardiac cell types, specifically by inhibiting cardiomyocyte contractile function, inducing macrophage inflammatory activation, stimulating microvascular inflammation and dysfunction, and promoting fibroblast transformation to a matrix-degrading phenotype (Frangogiannis, 2019; Packer and Kitzman, 2018). Inhibiting inflammatory cytokine activity may therefore serve as a key therapeutic target for SMLI to exert its anti-HF effects.

Restoration of vascular endothelial function: SMLI reduces ET-1 (MD = −4.72, *P* < 0.01) and increases NO (SMD = 2.63, *P* = 0.04). The endothelium—a single layer of cells lining the entire circulatory system—plays a critical role in regulating vascular tone, cell growth, inflammation, and thrombosis/hemostasis by producing numerous bioactive substances involved in these processes (Dmour et al., 2023). ET-1, a vasoconstrictive peptide primarily produced by endothelial cells and cardiomyocytes, contributes to HF pathophysiology through inducing cardiac hypertrophy and exerting pro-fibrotic and pro-inflammatory effects (Omland, 2019). A clinical evaluation of 115 patients with HF showed that ET-1 levels correlated with more advanced HF stages and reduced right ventricular function; furthermore, when incorporated into a multi-biomarker panel with other biomarkers, ET-1 may serve as a specific prognostic marker for HF (Gaggin et al., 2017). Endothelial cells protect arterial walls by releasing NO and prostacyclin. In patients with HF, endothelial dysfunction is mainly driven by increased production of superoxide free radicals and other oxidants in the vascular system. This oxidative stress state disrupts the balance between reactive oxygen species production and neutralization by endogenous antioxidant mechanisms, leading to direct NO inactivation and subsequent endothelial dysfunction. Beyond its role as a HF pathophysiological mechanism, endothelial dysfunction also acts as a prognostic indicator: it is associated with higher risks of hospitalization, heart transplantation, or death in patients with HF (Zuchi et al., 2020). Therefore, ET-1 and NO levels can be used to evaluate SMLI’s therapeutic effects on HF from the perspective of endothelial function.

#### 4.1.3 Clinical prognosis and safety assurance

##### 4.1.3.1 Improvement in functional prognosis

The 6MWTD was significantly increased (MD = 60.70, *P* < 0.01), indicating improved exercise endurance and an indirect improvement in quality of life. The 6MWT is well-established as a valid method for assessing prognosis in patients with various clinical conditions, including HF (Lars et al., 2005; Punekar et al., 2017; Rich, 2006). A meta-analysis by Andrew Coulshed further confirms that the 6MWT is a useful measure for evaluating physical fitness in patients with ischemic HF (Coulshed et al., 2023).

##### 4.1.3.2 Reduction in cardiovascular adverse events

The incidence of cardiovascular adverse events was significantly lower in the SMLI group (RR = 0.55, 95% CI: 0.37 to 0.83, *P* < 0.01), with no significant difference in the incidence of adverse reactions (e.g., dizziness, nausea, rash) relative to the control group (RR = 0.67, P = 0.09). This confirms SMLI’s efficacy-safety balance—an attribute of particular importance for patients with HF who require long-term management.

### 4.2 Mechanistic insights: multi-pathway regulation of SMLI in HF

#### 4.2.1 Activation of anti-inflammatory pathway

This meta-analysis provides convincing clinical evidence that SMLI exerts potent anti-inflammatory effects in patients with HF. One of our most important findings is that, compared with standardized treatment, combination therapy with SMLI significantly reduces the circulating levels of multiple core inflammatory markers in patients, including IL-6 (SMD = −2.36), TNF-α (SMD = −1.72), CRP (MD = −3.82), and hs-CRP (MD = −8.09). This comprehensive anti-inflammatory effect offers a key insight into the mechanism by which SMLI improves cardiac function. We propose that the anti-inflammatory activity of SMLI originates from the synergistic inhibition of multiple key inflammatory signaling pathways by its active components. The significant reductions in IL-6 and TNF-αlevels observed in the present study are highly consistent with the mechanism by which core components of Salvia miltiorrhiza (salvianolic acid A) inhibit the TLR2/4-MyD88-NF-κB signaling pathway (Dawuti et al., 2023). This pathway acts as a core hub mediating the production of proinflammatory cytokines. Bioactive components derived from Salvia miltiorrhiza block the activation of this pathway at the upstream level, thereby potently inhibiting the transcription and subsequent release of key proinflammatory cytokines in the downstream signaling cascade. (Zhang Z. et al., 2023). Meanwhile, our finding of significantly decreased levels of CRP and hs-CRP suggests that SMLI may act on a broader inflammatory network. TMP may play a pivotal role in this process. Studies have demonstrated that TMP can effectively inhibit the phosphorylation and activation of p38 mitogen-activated protein kinase (p38 MAPK) (Yu et al., 2015; Zhang H. et al., 2020), and the p38 MAPK pathway exerts important regulatory effects on liver-derived inflammatory markers such as CRP (Cavender MA et al., 2022). Therefore, the synergy between TMP and Salvia miltiorrhiza may constitute dual inhibition of the two major inflammatory pathways (NF-κB and MAPK), providing a plausible molecular explanation for the comprehensive anti-inflammatory effects observed in our meta-analysis.

In conclusion, the clinical data from the present study not only confirm that SMLI exerts a significant systemic anti-inflammatory effect but also identify this effect as one of the core mechanisms underlying its cardioprotective action. Through component complementarity, Salvia miltiorrhiza and TMP synergistically inhibit key inflammatory pathways, collectively alleviating persistent myocardial damage caused by chronic inflammation and thereby creating a favorable microenvironment for the improvement of LVEF and the recovery of cardiac function.

#### 4.2.2 Oxidative stress balance

The present study observed that SMLI treatment induced significant favorable changes in indices of vascular endothelial function in patients: plasma NO levels were significantly increased (SMD = 2.66), while ET-1 levels were significantly decreased (MD = −4.72). NO is a key regulator of endothelial function, and its bioavailability is strongly influenced by oxidative stress. Accordingly, the elevation of NO levels is a direct reflection of improved oxidative stress status. Based on this key finding, the active components in SMLI may restore the oxidant-antioxidant balance through a synergistic mechanism. Active components in Salvia miltiorrhiza (salvianolic acid B) have been shown to effectively reduce the accumulation of reactive oxygen species (ROS) and lipid peroxidation products (malondialdehyde [MDA]) (Xiao et al., 2020). Furthermore, components such as salvianolic acid A in Salvia miltiorrhiza can specifically promote the phosphorylation of endothelial nitric oxide synthase (eNOS), thereby increasing the production of NO—an agent with vasodilatory and antioxidant properties (Yang et al., 2011). This forms a positive feedback loop: reducing NO consumption on the one hand, and increasing NO synthesis on the other. TMP enhances and expands the antioxidant effects of Salvia miltiorrhiza through target complementarity. TMP not only inhibits the activity of xanthine oxidase (XO) at the source to reduce the generation of ROS such as superoxide (Zhang et al., 1994) but also promotes the synthesis of glutathione (GSH)—a crucial intracellular antioxidant—by upregulating the expression of SLC7A11 (Han et al., 2025), thereby enhancing the overall ROS scavenging capacity of cells. Improving oxidative stress is a key link in the mechanism by which SMLI exerts its effects.

#### 4.2.3 Inhibition of ferroptosis in myocardial cells

A key finding of the present meta-analysis is that SMLI combination therapy significantly reduces the levels of TnI, a core marker of myocardial injury (SMD = −1.97). The reduction in TnI clearly indicates that SMLI can effectively alleviate persistent cardiomyocyte injury, and ferroptosis—an important programmed cell death modality in HF—may be one of its targets. Based on this, we propose a novel hypothesis: inhibition of cardiomyocyte ferroptosis is an important mechanism underlying the cardioprotective effects of SMLI. This hypothesis is strongly supported by existing basic research. TMP has been shown to upregulate the expression of glutathione peroxidase 4 (GPX4), a key anti-ferroptotic protein, by activating the transcription factor Nrf2 (Han et al., 2025; Wu et al., 2024). GPX4 acts as the master switch in cellular defense against ferroptosis, its function is to scavenge toxic lipid peroxides, thereby blocking the iron-dependent lipid peroxidation cascade (Jiang et al., 2025). Components of Salvia miltiorrhiza may synergize with TMP to collectively enhance this pathway. Furthermore, Guanxinning Injection (GXN)—whose components are similar to those of SMLI—has been shown in HF models to exert cardioprotective effects by regulating the SLC7A11/GPX4 axis (Wang C. et al., 2023), providing circumstantial evidence that SMLI may exert similar effects. Thus, although direct clinical evidence remains to be validated, we infer that the reduction in TnI levels induced by SMLI may be partly attributed to its inhibition of cardiomyocyte ferroptosis via the Nrf2/SLC7A11/GPX4 signaling axis.

#### 4.2.4 Repair of vascular endothelial function

This meta-analysis provides clinical evidence that SMLI significantly improves vascular endothelial function in patients with HF. Our most direct finding is that SMLI potently reverses the core imbalance of endothelial dysfunction: it markedly increases the level of NO, a vasodilatory factor (SMD = 2.66), while reducing the level of ET-1, a potent vasoconstrictive factor (MD = −4.72). This effect of promoting vasodilation and inhibiting vasoconstriction provides a key pathophysiological basis for explaining the clinical benefits of SMLI. The results of the present study offer strong clinical support for the dual pharmacological effects of Salvia miltiorrhiza—enhancing NO production and inhibiting ET-1 synthesis. Specifically, danshensu from Salvia miltiorrhiza promotes the activation of endothelial nitric oxide synthase (eNOS) to increase NO generation, and salvianolic acid B inhibits endothelin-converting enzyme (ECE) to reduce ET-1 synthesis (Wei et al., 2023). More importantly, our analysis reveals another potential pathway through which SMLI improves endothelial function: inhibiting the overactivated renin-angiotensin system (RAS). The present study shows that SMLI significantly reduces PRA (MD = −4.60). This is highly consistent with the well-documented effects of TMP—inhibiting renin activity and reducing the production of Ang II (Qin et al., 2011; Zhang B. et al., 2020). Since Ang II is a key factor contributing to endothelial dysfunction and oxidative stress, TMP, by inhibiting the RAS axis, synergizes with Salvia miltiorrhiza to collectively improve vascular endothelial health from multiple levels, laying a solid vascular foundation for the recovery of cardiac function.

#### 4.2.5 Reversal of myocardial fibrosis

The present meta-analysis reveals the comprehensive ameliorative effects of SMLI on cardiac structure and function in patients with HF. We found that SMLI treatment not only significantly enhances cardiac pumping function—such as CO (MD = 0.85) and SV (MD = 5.80)—but more importantly, it markedly improves cardiac structural indices, including reducing LVESD (MD = −4.72) and LVESV (MD = −13.25). Based on these key clinical findings, we propose that the active components in SMLI counteract myocardial fibrosis by synergistically regulating collagen metabolism. Salvianolic acid A from Salvia miltiorrhiza may inhibit the Smad2/3 signaling pathway of transforming growth factor-β1 (TGF-β1)—a core profibrotic factor—thereby reducing the phosphorylation and nuclear translocation of Smad3. This downregulates the gene expression of type I and type III collagen at the source and inhibits the synthesis of new collagen fibers (Wu et al., 2014). Meanwhile, TMP may promote the degradation of abnormally deposited collagen networks by upregulating the activity of enzymes such as matrix metalloproteinase-9 (MMP-9) (Huang et al., 2018). This dual regulatory effect—inhibiting collagen synthesis and promoting collagen degradation—helps restore the normal ratio of cardiomyocytes to extracellular matrix in myocardial tissue and improves myocardial compliance (Kong et al., 2020).

#### 4.2.6 Inhibition of cardiomyocyte apoptosis

The present meta-analysis confirms that SMLI significantly improves LVEF (MD = 5.62) and alleviates myocardial injury (TnI, SMD = −1.97) in patients with HF. The long-term maintenance of cardiac function fundamentally depends on the number of functional cardiomyocytes. Thus, in addition to mitigating acute injury, inhibiting the apoptotic process that leads to the chronic loss of cardiomyocytes represents a key potential mechanism underlying the sustained therapeutic effects of SMLI. Studies have shown that active components in Salvia miltiorrhiza can regulate the balance of Bcl-2 family proteins—upregulating the anti-apoptotic protein Bcl-2 and downregulating the pro-apoptotic protein Bax—thereby inhibiting the activation of the Caspase signaling pathway and effectively blocking cardiomyocyte apoptosis (Wang et al., 2017). An injection with the same composition as SMLI has also been shown in cellular models to significantly regulate the expression of key apoptosis-related proteins such as Bcl-2, Bax, and Caspase-3 (Wu, Z. X. et al., 2022). Based on these findings, we propose that the improvements in LVEF and reduction in TnI observed with SMLI may be partly attributed to its inhibition of cardiomyocyte apoptosis via the aforementioned pathways, thereby helping to maintain the number and functional integrity of cardiomyocytes.

### 4.3 Evidence positioning: comparison with existing studies

The innovative value and limitations of the present study should be assessed against the backdrop of existing evidence. First, individual small-sample studies (Li et al., 2024; Lin, 2024) have previously reported that SMLI can improve LVEF and BNP levels in patients with HF, but most of these studies had a sample size of <100 cases, resulting in limited robustness of their conclusions. Via a large-sample meta-analysis encompassing 3,077 patients, the present study is the first to quantitatively validate the therapeutic efficacy of SMLI, providing higher-level evidence for the optimization of clinical regimens. Second, existing meta-analyses on TCM injections for HF (e.g., Shenfu Injection, Huangqi Injection) have mostly focused on improvement in cardiac function as the primary outcome (Cao et al., 2022; Wu, Y. et al., 2022). In contrast, the present study systematically incorporated intermediate endpoints—including inflammation, vascular endothelium, and neuroendocrinology—and verified that SMLI reduces cardiovascular adverse events. The inclusion of this endpoint extends the clinical value of SMLI beyond symptom relief to prognosis improvement, addressing the prognostic evidence gap in the use of TCM injections for HF. Finally, in the present study, SMLI was administered in combination with conventional Western medical therapy. Results demonstrated that SMLI further enhances therapeutic efficacy without increasing the risk of adverse reactions. This indicates that SMLI is not an alternative therapy for HF but a complementary therapy, which can serve as a valuable adjunct to the standardized treatment of HF.

### 4.4 Constraints and limitations

The limitations of the present study should be objectively acknowledged to preclude overinterpretation of the results. First, the overall quality of the included studies is relatively modest. All 32 included studies were from Chinese journals; of these, 14 did not specify the method of random sequence generation, and none reported allocation concealment or blinding. This may introduce selection and performance bias, thereby compromising the validity of the results. Furthermore, no international multicenter studies were included, resulting in substantial regional bias; caution is therefore warranted when extrapolating the results to non-Asian populations. Second, the core outcomes exhibit substantial heterogeneity, and some lack adequate evidence strength. Substantial heterogeneity was observed across core outcomes (e.g., LVEF: I^2^ = 93.3%; BNP: I^2^ = 94.3%), and the sources of heterogeneity for outcomes such as LVEF and inflammatory markers (e.g., patients’ baseline NYHA class, comorbidity differences) remain incompletely elucidated. Moreover, the number of studies included for outcomes such as 6MWTD and TnI is small, and the stability of the evidence needs further validation. Third, the long-term efficacy and dose-response relationship of SMLI remain unclear. The treatment duration in the included studies mostly ranged from 10 days to 6 months, with a paucity of long-term follow-up data beyond 1 year; this precludes evaluation of SMLI’s impact on long-term mortality and readmission rates in patients with HF. Finally, there is a risk of publication bias for some outcomes. Egger’s test indicated the presence of publication bias for NT-proBNP (*P* = 0.004) and ET-1 (*P* = 0.024), which may be associated with the common phenomenon of publication bias—whereby studies with positive results are more likely to be published. Additional data from future studies are required for further validation.

### 4.5 Clinical implications and future directions

Based on the findings of the present study, the following principles can guide the clinical application of SMLI in the treatment of HF: First, regarding the target population: SMLI is recommended for use in patients with HF of New York Heart Association (NYHA) functional classes II–IV, particularly those with a high inflammatory burden (CRP >5 mg/L), endothelial dysfunction (ET-1 or reduced NO levels), or increased PRA. Second, concerning the treatment regimen: administration of SMLI via intravenous infusion is recommended in addition to conventional Western medical therapy for HF. Third, with respect to safety monitoring: although the incidence of adverse reactions to SMLI is low, attention should still be paid to events such as hypotension and rash. Importantly, a detailed allergy history should be evaluated prior to the initiation of SMLI treatment.

### 4.6 Concluding

This meta-analysis demonstrates that SMLI, when administered in combination with conventional therapy, significantly enhances cardiac function, inhibits inflammatory responses, repairs vascular endothelial dysfunction, and reduces cardiovascular adverse events in patients with HF, with a favorable safety profile. Its therapeutic effects are likely mediated by mechanisms including inhibiting inflammation, suppressing cardiomyocyte ferroptosis and apoptosis, regulating oxidative stress, and selectively modulating the RAS. Despite inherent limitations—such as the modest methodological quality of included studies and substantial regional bias—this study nevertheless provides high-level evidence supporting the clinical application of SMLI in HF treatment. Moving forward, well-designed, high-quality studies are warranted to further validate its long-term efficacy and elucidate its underlying mechanisms, thereby facilitating the standardization and precision of integrated traditional Chinese and Western medicine in the management of HF.

## Data Availability

The original contributions presented in the study are included in the article/Supplementary Material, further inquiries can be directed to the corresponding author.
