# Supplementary material for: Efficacy and safety of Salvia miltiorrhiza and ligustrazine injection for heart failure: a systematic review and meta-analysis
Source: Front Pharmacol. 2026 Feb 9;17:1692629. doi: 10.3389/fphar.2026.1692629 (PMC12926771; doi:10.3389/fphar.2026.1692629)
Supplement: Supplementary file 1 [file Supplementaryfile1.docx]

Supplementary materials

## Table S1. The search strategies for all databases.

| **The search strategy for PubMed** | |
| --- | --- |
| **Number** | **Search terms** |
| #1 | Cardiac Failure[MeSH Terms] |
| #2 | Heart Failure[MeSH Terms] |
| #3 | ((((heart failure[Title/Abstract]) OR (cardiac failure[Title/Abstract])) OR (CHF[Title/Abstract])) OR (chronic heart failure[Title/Abstract])) OR (Acute heart failure[Title/Abstract]) |
| #4 | #1 OR #2OR #3 |
| #5 | Search:((((Salvia Ligustrazine[Title/Abstract]) OR (Danshen Chuanxiongqin[Title/Abstract])) OR (Radix Salivae Miltiorrhizae ligustrazine[Title/Abstract])) OR (danshenchuanxiong[Title/Abstract]) |
| #6 | #4 AND #5 |
| **The search strategy for Embase** | |
| **Number** | **Search terms** |
| #1 | 'heart failure':ti,ab,kw OR 'cardiac failure':ti,ab,kw OR 'chronic heart failure':ti,ab,kw OR 'acute heart failure':ti,ab,kw |
| #2 | 'salvia ligustrazine':ti,ab,kw OR 'danshen chuanxiongqin':ti,ab,kw OR 'radix salivae miltiorrhizae ligustrazine':ti,ab,kw OR danshenchuanxiong:ti,ab,kw |
| #3 | #1 OR #2 |
| **The search strategy for Cochrane Library** | |
| **Number** | **Search terms** |
| #1 | (Heart Failure):ti,ab,kw OR (Cardiac Failure):ti,ab,kw OR (CHF):ti,ab,kw OR (chronic heart failure):ti,ab,kw OR (Acute heart failure):ti,ab,kw |
| #2 | (Salvia Ligustrazine):ti,ab,kw OR (Danshen Chuanxiongqin):ti,ab,kw OR (Radix Salivae Miltiorrhizae ligustrazine):ti,ab,kw OR (danshenchuanxiong):ti,ab,kw |
| #3 | #1 OR #2 |
| **The search strategy for WOS** | |
| **Number** | **Search terms** |
| #1 | (((AB=(heart failure)) OR AB=(cardiac failure)) OR AB=(chronic heart failure)) OR AB=(acute heart failure) |
| #2 | (((AB=(salvia ligustrazine)) OR AB=(danshen chuanxiongqin)) OR AB=(radix salivae miltiorrhizae ligustrazine)) OR AB=( danshenchuanxiong) |
| #3 | #1 OR #2 |
| **The search strategy for CNKI** | |
| (SU = '心衰' OR SU = '心力衰竭' OR SU = '心水' OR SU = '慢性心衰' OR SU = '急性心衰') AND (SU= '丹参川芎嗪'OR OR SU = '丹参川芎嗪注射液') | |
| **The search strategy for WanFang** | |
| 主题: ("心衰" OR "心力衰竭" OR "慢性心力衰竭"OR "慢性心力衰竭"OR "心水") and 主题: ("丹参川芎嗪"OR "丹参川芎嗪注射液") | |
| **The search strategy for VIP** | |
| ((M=心衰 OR 心力衰竭 OR 慢性心力衰竭 OR 急性心力衰竭 OR 心水) OR (R=心衰 OR 心力衰竭 OR 心水)) AND ((M=丹参川芎嗪 OR 丹参川芎嗪注射液 ) OR (R=丹参川芎嗪 OR 丹参川芎嗪注射液)) | |
| **The search strategy for CBM** | |
| ((("心衰"[常用字段:智能] OR "心力衰竭"[常用字段:智能] OR "慢性心力衰竭"[常用字段:智能] OR "急性心力衰竭"[常用字段:智能] OR "心水"[常用字段:智能])) AND (("丹参川芎嗪"[常用字段:智能] OR "丹参川芎嗪注射液"[常用字段:智能]))) | |


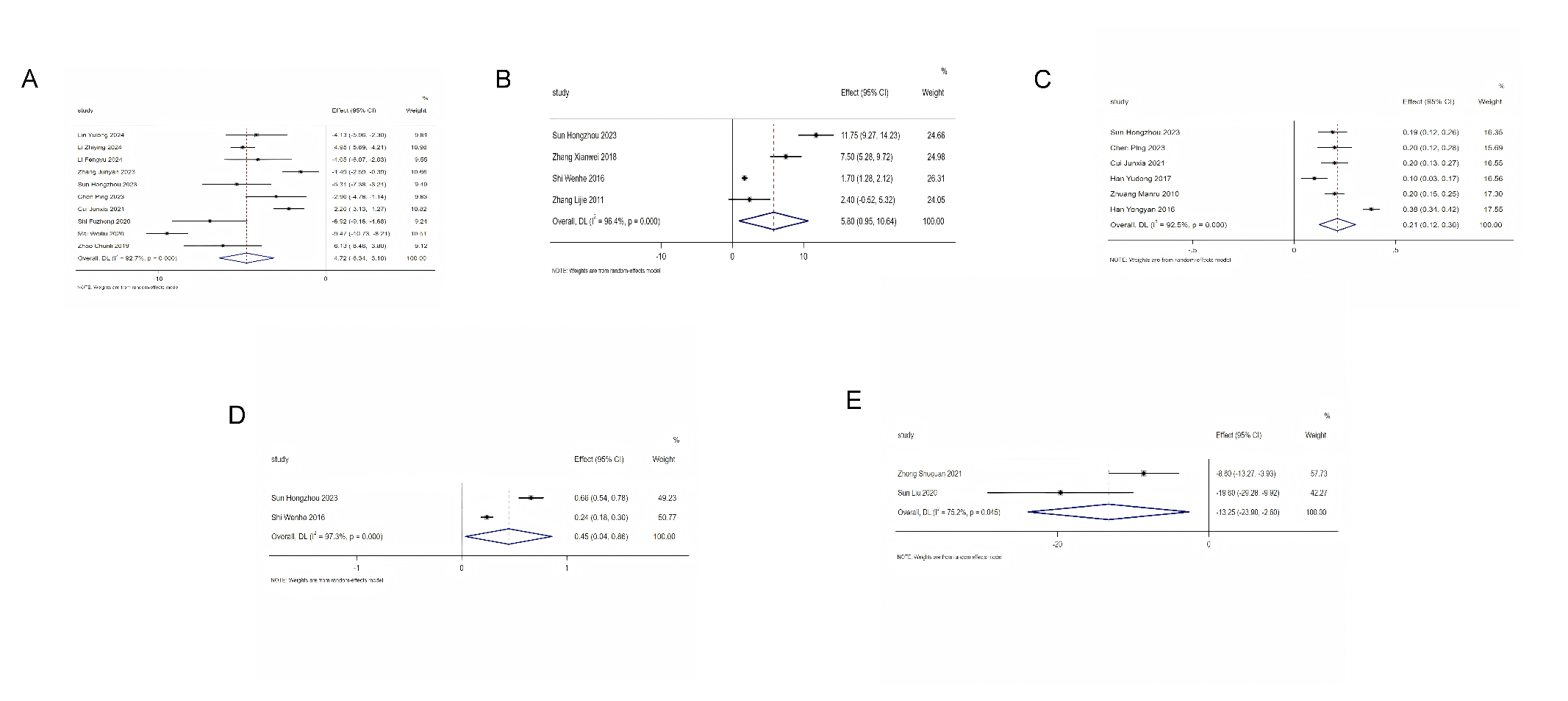


Figure S1 Forest plot. A:LVESD ,B:SV, C:E/A, D:CI, E:LVEDV





Figure S2 Sensitivity analysis. A:LVESD ,B:SV, C:E/A, D:CI, E:LVEDV


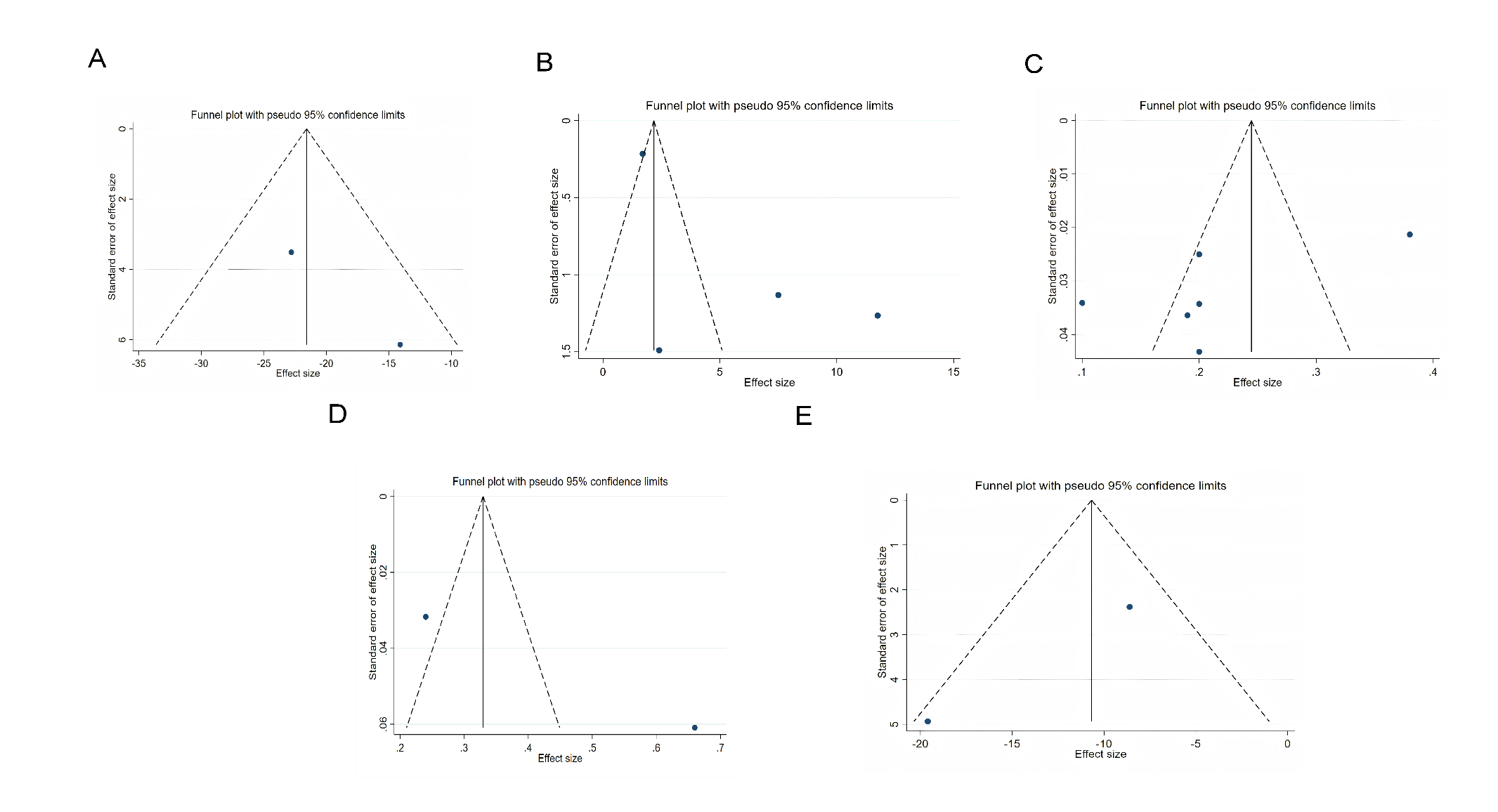


Figure S3 Funnel plot. A:LVESD ,B:SV, C:E/A, D:CI, E:LVEDV
